# Techno-Economic Assessment of a Scaled-Up Meat Waste Biorefinery System: A Simulation Study

**DOI:** 10.3390/ma12071030

**Published:** 2019-03-28

**Authors:** Oseweuba Valentine Okoro, Zhifa Sun, John Birch

**Affiliations:** 1 Department of Physics, University of Otago, P.O. Box 56, Dunedin 9054, New Zealand; 2 Department of Food Science, University of Otago, P.O. Box 56, Dunedin 9054, New Zealand; john.birch@otago.ac.nz

**Keywords:** meat waste biorefinery, economic performance, environmental performance, simulation study, optimization

## Abstract

While exports from the meat industry in New Zealand constitute a valuable source of foreign exchange, the meat industry is also responsible for the generation of large masses of waste streams. These meat processing waste streams are largely biologically unstable and are capable of leading to unfavourable environmental outcomes if not properly managed. To enable the effective management of the meat processing waste streams, a value-recovery based strategy, for the complete valorisation of the meat processing waste biomass, is proposed. In the present study therefore, a biorefinery system that integrates the biomass conversion technologies of hydrolysis, esterification, anaerobic digestion and hydrothermal liquefaction has been modelled, simulated and optimized for enhanced environmental performance and economic performance. It was determined that an initial positive correlation between the mass feed rate of the waste to the biorefinery system and its environmental performance exists. However, beyond an optimal total mass feed rate of the waste stream there is a deterioration of the environmental performance of the biorefinery system. It was also determined that economies of scale ensure that any improvement in the economic performance of the biorefinery system with increasing total mass feed rate of the waste stream, is sustained. The present study established that the optimized meat waste biorefinery system facilitated a reduction in the unit production costs of the value-added products of biodiesel, biochar and biocrude compared the literature-obtained unit production costs of the respective aforementioned products when generated from stand-alone systems. The unit production cost of biogas was however shown to be comparable to the literature-obtained unit production cost of biogas. Finally, the present study showed that the optimized meat processing waste biorefinery could achieve enhanced economic performance while simultaneously maintaining favourable environmental sustainability.

## 1. Introduction

Biorefineries are systems that integrate different conversion technologies to generate multiple useful products while using biomass as a renewable feedstock resource [1,2]. Based on this definition, a biorefinery may be considered as being similar to a typical crude oil refinery that employs crude oil as the feedstock to produce multiple useful products [1]. The renewability of biomass as a resource provides an opportunity for the development of sustainable pathways for the production of biofuels and biochemicals. In line with current interest in the employment of the biorefinery paradigm as a viable approach to counter challenges of resource depletion and global warming [1,2], the present study has explored the viability of utilizing meat processing waste as a sustainable biomass resource for a biorefinery system. In this research, the country of New Zealand is specified as the case study with meat processing waste investigated as a biomass feedstock that is sufficient to demonstrate the sustainability of the proposed biorefinery system. Meat processing waste has been specified as a viable biomass resource in New Zealand since according to [1,3,4]:

- Significant masses of meat processing wastes are generated annually from meat processing related activities in New Zealand.
- Meat processing wastes constitute major management challenges due to their unfavorable impacts on land, water and air when improperly managed (Richard Stapel, Waste solutions- New Zealand, personal communication, 2015).
- Major technologies such as composting and incineration, employed in waste management in New Zealand, are characterized by several limitations such as requirements for large land area and large mass of bulking material, difficulty in dewatering the waste and the generation odorous air, dust and other emissions.
- Most importantly, meat processing wastes may serve as biomass resources that are available in the absence of associated costs of cultivation, harvesting or agricultural land that typically characterizes plant-sourced biomass.
- There is therefore a clear opportunity to improve the economic performance of existing meat processing plants, via the recovery of valuable products from the meat processing waste which may generate additional revenue streams when sold.

In recognition of the aforementioned benefits of utilizing meat processing waste as a sustainable biomass resource, previous studies have experimentally explored the untapped potential of utilizing meat processing waste biomass as a viable biorefinery feedstock [3,4,5,6,7,8]. These previous studies initially appreciated that the utilization of meat processing wastes as biorefinery feedstocks may present some challenges as a result of its typically high moisture content. This is because most biomass conversion technologies favour dry biomass as feedstock for bioenergy and biochemicals production. A review of existing biomass technologies was therefore undertaken in [1] to enable the proper screening of the possible biomass technologies and identify those technologies that would be sufficient to facilitate the transformation of high moisture meat processing waste feedstocks. Biomass conversion technologies of hydrolysis-esterification, anaerobic digestion and hydrothermal liquefaction conversion technologies were consequently selected as the preferred technologies [3,4,5,6,7]. In the studies undertaken in [1,3,4,5], the conversion of the lipids present in the meat processing waste of high moisture dissolved air flotation sludge to biodiesel was considered crucial to the functionality of the proposed biorefinery since there was a risk of anaerobic digestion (AD) failure if the high lipid concentrations in the inlet stream to the AD process was maintained. Crucially the need to eliminate the high energy requirement that would characterize any preliminary drying or dewatering operation, prior to lipid extraction necessitated the approach of producing FAs via the so called ‘in-situ hydrolysis’ pathway. As stated earlier above moisture-favouring biomass conversion technologies of namely AD and hydrothermal liquefaction were integrated in the proposed biorefinery since they guarantee biofuel and biochemical production respectively in the absence of the need for preliminary dewatering operations. Furthermore, the integration of the hydrothermal liquefaction process as a ‘terminal’ biomass conversion technology eliminates the need for the incorporation of further downstream sterilization steps due to the high temperature and high pressure conditions typically imposed [1,6,7,8].

These previous studies were therefore able to demonstrate the possibility of generating useful products of biodiesel, biocrude, biochar and biogas from meat processing waste, via the employment of biomass conversion technologies of hydrolysis-esterification, AD and hydrothermal liquefaction conversion technologies respectively [3,4,5,6,7].

Although the aforementioned biomass conversion technologies were shown to be feasible when assessed via laboratory scale experiments, the viability and performance of the large-scale integration of these biomass conversion technologies is yet to be assessed. In this study therefore, the environmental performance and economic performance of a large-scale biorefinery system operating in steady state, albeit simplified, will be investigated. The novelty of the study is emphasized by the unconventionality of both the feedstocks employed and the complexity of the processing scheme of the proposed biorefinery. Given that the biorefinery system is composed of selected biomass conversion technologies that have been extensively investigated in previous studies [3,4,5,6,7], the experimental results generated from these studies will serve as an invaluable input data resource for the simulation study.

## 2. Process Modelling and Simulation Methodology

### 2.1. Process Modelling Software

The biorefinery system has been modelled and simulated using the commercially available ASPEN (Advanced System for Process Engineering, version10) plus. ASPEN plus is employed in process design, modelling and simulation [9]. ASPEN plus facilitates the resolution of process flowsheets by invoking sequential modular and equation oriented modelling strategies [10]. Sequential modular modelling and equation oriented modelling strategies enable ASPEN plus to resolve a large number of unit operation blocks sequentially and solve a large number of equations (e.g., energy balance and mass balance equations) simultaneously [10,11].

### 2.2. Process Description

**Figure 1:**
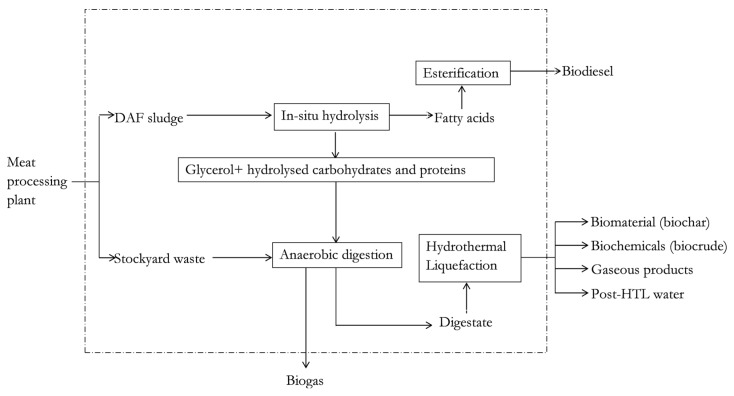
Biorefinery system using meat processing waste as the feedstock. DAF denotes dissolved air flotation sludge, HTL denotes hydrothermal liquefaction.

Figure 1 shows the schematic illustration of the biorefinery composed of the selected biomass conversion technologies. The system boundaries have been specified using the dashed lines in Figure 1. Figure 1 highlights the generation of the useful product streams, namely the biogas (gaseous biofuel), biodiesel (liquid biofuel), biochar (biomaterial for soil enhancement) and biocrude (biochemical source or liquid biofuel) from the meat processing waste streams of dissolved air flotation (DAF) sludge and stockyard (SY) waste. It is assumed that the biorefinery system operates for 300 days/year (7200 h/year), all exit hot streams are cooled to 25 °C to enhance opportunities for heat recovery and there is a base case availability of 1000 tonnes of DAF sludge per day (or 41.7 tonnes/h). The base case mass feed rate of the SY waste will be determined from the mass feed rate of the DAF sludge during the simulation run. This is because the mass feed rate of the DAF sludge directly influences the mass feed rate of wet hydrolysed DAF sludge (WHDS), from the in-situ hydrolysis processing of DAF sludge, employed for biomethane production as discussed in [6].

#### 2.2.1. Catalysed In-Situ Hydrolysis and Esterification of DAF Sludge

As shown in Figure 1, biodiesel is generated using the catalysed in-situ hydrolysis and esterification pathway. The generation of biodiesel from DAF sludge via the in-situ hydrolysis pathway has been simulated and discussed extensively in [5].

Briefly, in-situ lipid hydrolysis processing of the wet DAF sludge (92 wt.% wet basis) is achieved under the imposed reaction conditions of temperature, pressure and catalyst load of 92.5 °C, 1 atm and 0.09216 kg-Dowex 50 WX2 resin/kg-wet fresh DAF sludge respectively according to a previous study [3]. DAF sludge lipids (DSL) have been modelled using Zong’s Fragment-based approach as extensively discussed elsewhere [12]. The protein content and carbohydrate content of DAF sludge are modelled as L-phenylalanine [13] and glucose [14] respectively. Other chemical inputs such as methanol, water and glycerol employed in the simulation study were obtained from the databank of the chemical property library in ASPEN plus^®^ V10. It has been proposed that DSL hydrolysis reaction occurs as follows,

(1) $$\mathrm{DSL}\;+\;3H_{2}O\overset{\mathrm{catalyst}}{\to }\mathrm{Glycerol}\;+\;3\mathrm{DFA}$$

where DFA represents DAF sludge fatty acids.

This DSL hydrolysis reaction has been simulated using a simple stoichiometric reactor block in ASPEN plus, with a 98% conversion of the DSL imposed [15]. This preliminary simulation study assumes complete recoverability of the Dowex 50 WX2 resin beads for simplicity. For the esterification reaction, the DAF sludge fatty acid, DFA, is modelled as oleic acid due to reasons discussed in Reference [5], with methylation achieved using excess methanol [5,16]. The esterification reaction is homogeneous first-order reaction occurring under the action of 0.0354 kg of solid 12-tungstophosphoric acid (as the catalyst) per kg of DAF fatty acid and supported on silica [16]. The esterification reaction is modelled according to the following reaction equation,

(2) $$\mathrm{DFA}+\;\mathrm{Methanol}\overset{\mathrm{catalyst}}{\to }\mathrm{DSME}\;+\;\mathrm{water}$$

where, DSME denotes the DAF sludge methyl ester.

#### 2.2.2. Anaerobic Co-Digestion Process of WHDS and SY

The wet hydrolysed dissolved air flotation sludge (WHDS) residue is subjected to an anaerobic digestion process while utilizing the stockyard (SY) waste stream as a co-digestion substrate to enhance the useful biomethane potential via the introduction of established synergizing effects [6]. According to Okoro et al. [6] the preferred mix ratio of the mass of stockyard waste to the mass of WHDS is 4 to 1, on a volatile solid basis for a favourable biomethane yield from an anaerobic digestion process occurring at a temperature condition and pressure condition of 37 °C and 1 atm respectively. To model the anaerobic digestion process, it is recognized that the AD process is an exceptionally complex biological process defined by a series of multi-step, overlapping processes that are dependent on numerous factors such as the microbial population growth and decline, nutrient content, pH value, temperature and inoculum substrate ratio [1,6]. Several simplifying specifications were therefore imposed to enable the successful simulation of the anaerobic digestion process. Firstly, it was assumed that equilibrium states exist during the degradation process, with the biogas potential for the specified substrate mix calculated by minimizing the Gibbs free energy (*G*) of the system while simultaneously satisfying mass balance and energy balance constraints. This assumption is justified since the degradation reactions that occur during the anaerobic digestion process always attain rates such that the reacting species are close to their respective equilibrium states (∆*G_total_* = 0) [17]. The total Gibbs free energy *G_total_* for the AD system with *N* species is expressed by the following equation [18,19],

(3) $$G_{total}=\sum_{i=1}^{N}n_{i}G^{0}{}_{f.i}+\sum_{i=1}^{N}\left(n_{i}RT\ln\left(\frac{f_{i}}{f_{i}{}^{o}}\right)\right)$$

where for gas phase species, *f_i_*^0^ is equal to 1 bar and,

(4) $$f_{i}=\theta_{i}y_{i}P$$

and for liquid phase species,

(5) $$\frac{f_{i}}{f_{i}{}^{o}}=a_{i}$$

In the above equations, *f_i_*^0^ denotes the standard molar fugacity of species *i*, *G_f,i_*^0^ is the Gibbs free energy of formation of species *i* at standard pressure of 1 bar; θ*_i_* is the fugacity coefficient of species *i*; *R* is the universal gas constant specified as 8.314 J/mol·K, *T* is the temperature in K, *a_i_* is the activity of species *i, y_i_* is the mole fraction vapour of specie *i*. ASPEN plus is able to minimise the objective function (*G_total_*) by setting the differentiated equation (with respect to *n_i_*) and subsequently solving for *n_i_*. Fugacities, activities and fugacity coefficients are estimated by ASPEN plus using thermodynamic property methods. A similar approach is taken when assessing both complex chemical equilibria and complex phase equilibria for all chemical species. Secondly, the experimentally determined biomethane potential expressed as the volume of biomethane produced in mL per unit mass of volatile solids in g in [6] has been employed as the biomethane potential of the anaerobic process in the present simulation study. This constitutes a common methodology that has been extensively applied to process simulations of AD processes [20,21,22,23,24]. Thirdly to specify the fraction of the available organic substrates that can be degraded anaerobically for biomethane production, the anaerobic biodegradability of the substrate mixture is another important parameter important for a successful simulation of the anaerobic co-digestion process. The anaerobic biodegradability (*X_d_*) of a substrate mixture is calculated by comparing the experimentally determined biomethane potential (BMP_e_) in mL/g-VS_added_ from the co-substrate mixture with the associated theoretical maximum biomethane potential (BMP_t_) in mL/g-VS_added_ determined using Equation (6) below,

(6) $$X_{d}=\frac{{\mathrm{BMP}}_{e}}{{\mathrm{BMP}}_{t}}$$

This anaerobic biodegradability (*X_d_*) is numerically equivalent to the mass fraction of the substrate volatiles available that can be degraded under the anaerobic conditions. The experimentally determined biomethane potential (BMP_e_) can be obtained from the results presented in Reference [6] while the theoretical maximum biomethane potential (BMP_t_) is calculated using Buswell’s relation, which is based on the elemental content of the substrates, as follows [25,26,27,28],

(7) $${\mathrm{BMP}}_{t}=\frac{22400\left[\left(\frac{c}{2}\right)+\left(\frac{h}{8}\right)-\left(\frac{o}{4}\right)-\left(\frac{3n}{8}\right)-\left(\frac{s}{4}\right)\right]}{\left(12c+h+16o+14n+32s\right)}$$

where *c*, *h*, *o*, *n* and *s* are subscripts representing the number of atoms present in organics with a chemical formula of C*_c_*H*_h_*O*_o_*N*_n_*S*_s_* which represents the substrate mixture being degraded anaerobically. The chemical formulae of stockyard waste and wet hydrolysed DAF sludge residue were estimated to be C_297_H_504_N_18_O_169_S and C_48_H_79_N_2_O_17_S respectively [6].

#### 2.2.3. Hydrothermal Liquefaction Process of Digestate

In previous studies, the hydrothermal liquefaction process was shown to be characterised by the presence of equilibrium states [29,30]. Variations of the reaction equilibrium conditions were demonstrated as having implications on the biochar yield and biocrude yield [29,30]. Recognising the complexity of the hydrothermal liquefaction process, the yields of the major products namely biocrude, biochar, gas phase and post-HTL water were also predicted using the non-stoichiometric chemical equilibrium reactor model of the ASPEN plus. The non-stoichiometric chemical equilibrium reactor model was employed since there is currently no consensus among researchers with respect to a universal chemical reaction (or series of chemical equations) that can completely and efficiently describe the HTL process for different biomass feed streams. The non-stoichiometric chemical equilibrium reactor model employs the Gibbs free energy minimisation principle discussed in Section 2.2.2 above. Post-HTL water and biochar containing mainly water (99.4 wt.%) and mainly ash (75.5 wt.%) [7], were modelled as water and ash respectively. For simplicity the gas product was modelled as a mixture of CO_2_ and N_2_ gases [31,32,33]. The biocrude has been modelled as a liquid fuel, heavy fraction of petroleum, in the ASPEN plus to limit possible convergence issues. Convergence issues may arise due to the complexity of composition of biocrude and the complexity of the integrated biorefinery system. This assumption is justified by the similarities between the biocrude and the heavy fraction of petroleum crude as discussed in previous studies [1,7,34]. Thus, the thermodynamic properties of the heavy petroleum crude predicted using enhanced American Petroleum Institute (API) correlations and the algorithms of ASPEN plus are used to approximately represent those of the biocrude. As a warning, however, it is recognised that although biocrude exhibits some fuel-like and compositional similarities to petroleum crude, biocrude cannot be considered as an exact analogue of petroleum crude.

To determine the appropriate estimation model of thermodynamic properties of substance streams in the biorefinery system simulated, the decision steps for thermodynamic property selection outlined by Carlson [35], Edwards [36] and in ASPEN’s operation manual [37] were reviewed. A review of the aforementioned guides indicate that the non-random two-liquid (NRTL) is sufficient to predict corresponding phase equilibrium compositions of chemical species at low pressures (<1000 kPa) and moderate temperatures (2–202 °C) and the Redlich–Kwong–Aspen equation is sufficient for higher pressures (>1000 kPa) and higher temperatures (>202 °C). The NRTL-Redlich-Kwong EOS property method was therefore employed in the simulation of the biorefinery process.

## 3. Energetic Analysis, Environmental and Economic Performance Assessments

### 3.1. Energetic Analysis and Environmental Performance

In the present study, the energetic assessment of the proposed biorefinery system, was undertaken using ASPEN energy analyser^®^ V.10. The pinch analysis technique in the ASPEN energy analyser^®^ V.10 has been employed to conduct heat integration for the proposed biorefinery system. The ‘hot’ and ‘cold’ streams in the biorefinery system have been determined to investigate what proportion of the heating and cooling requirements of the biorefinery system can be satisfied internally. A minimum allowable temperature difference, 10 °C, between the ‘hot’ and ‘cold’ streams at ‘pinch point’ in the heat exchanger system has been specified [38]. ASPEN energy analyser^®^ V.10 has also been used in calculating the area and number of shell and tube heat exchangers required. In addition to the total heating and cooling duty, electrical duties for pumps and mechanical stirrers have also been determined. While the electrical duty required by the high pressure pump is readily estimated in ASPEN plus, ASPEN plus assumes complete mixing between the reactants. This implies that the estimates for the electrical energy duty required for mixing in the reactor vessels are not provided by ASPEN plus. To further improve the realism of the model estimates, the electrical duties required by mechanical stirrers were estimated. It is recognised that the electrical duty of mechanical stirrers in liquid-solid systems is dependent on several parameters such as the type of impeller, nature of the mixture and important reactor geometric parameters such as its volume and capacity [39]. To however simplify the analysis, it is assumed that the electrical power required for the stirrers in each reactor can be estimated by utilising the average specific mixing power of mechanical systems in large-scale solid-liquid system specified as 0.5 kW/m^3^ of mixture [39]. The electrical duty required by each mechanical stirrer can therefore be estimated from the knowledge of the volume of the mixture present in each reactor. A similar approach was employed in [5]. The summation of the determined total electrical duty and the total heating duty were therefore determined to obtain the total energy duty requirement of the biorefinery system.

The energy potential of the proposed biorefinery system is estimated from the knowledge of the yield and higher heating values (HHVs) of the energy dense product streams. In this study the NER is utilised as a simple environmental performance index, since its sufficiency as a surrogate measure of environmental sustainability was previously demonstrated [40,41]. The NER of the biorefinery system is defined as follows,

(8) $$\mathrm{NER}=\frac{\sum_{j}^{n}\left({\text{HHV}}_{j}\times P_{j}\right)}{\left(\left[\frac{\sum_{i}^{S}E_{s-E,i}+E_{p-E}}{\eta_{H-E}}\right]+\frac{\sum_{i}^{N}E_{H-f,i}}{\eta_{C-H}}\right)}$$

where HHV*_j_* represents the higher heating value of the *j*th energy dense product (the HHV of biodiesel, biogas and biocrude have been determined to be 39,800 [4], 20,900 [6] and 36,700 kJ/kg [7,34] respectively; *P_j_* represents the production capacity of the *j*th energy dense products of biodiesel, biocrude and biogas, in kg/h, the other useful product of biochar is not considered as an energy dense fuel due to its low HHV of 4580 kJ/kg as discussed in Reference [6]; *E_H−f,i_* denotes the input thermal energy in kJ/h for the *i*th major equipment; *E_s−E,i_* represents the electrical energy required by the *i*th stirrer; *E_p−E_* represents the electrical energy required by the HTL high pressure pump; η*_C−H_* and η*_H−E_* represent the thermal efficiencies of conversion of chemical energy (biodiesel, biocrude and biogas) to thermal energy and electrical energy, respectively, which are specified as 0.9 and 0.47 [42,43,44]; *n, S* and *N* are the number of energy dense products, number of reactor stirrers and number of equipment. For countries such as New Zealand where electricity can be generated from renewable sources such as hydropower, the electrical energy term will be ignored. This is because renewable energy sources are not associated with unfavorable environmental impacts and unpleasant sustainability concerns. Two cases, electricity generation from fossil sources (case A) and electricity generation from renewable sources (case B) will therefore be assessed in this study. For both cases A and B, an NER value greater than 1 is indicative of a favorable environmental performance [45].

### 3.2. Economic Assessment of the Biorefinery System

To investigate the economic performance of the biorefinery system several economic assessment metrics have been initially considered. These economic assessment metrics are namely, the production cost per unit higher heating values of the useful products, (*C_h,j_*), in $US/MJ in year *j*, the production cost per unit price of the useful products (*C_p,j_*) (dimensionless) in year *j* and the production cost per unit mass of useful products (*C_m,j_*) in $US/tonne, in year *j*. These economic assessment metrics are calculated using the following equations, respectively,

(9) $$C_{h,j}=\frac{C_{T,j}}{\sum_{i=1}^{n}m_{p,i}{\text{HHV}}_{i}}$$

(10) $$C_{p,j}=\frac{C_{T,j}}{\sum_{i=1}^{n}m_{p,i}p_{i}}$$

(11) $$C_{m,j}=\frac{C_{T,j}}{\sum_{i=1}^{n}m_{p,i}}$$

where in Equations (9)–(11), *C_T,j_* is the total annual cost of the biorefinery system in $US in year *j*; *m_p,i_* is the mass of the *i*th useful product of biochar, biodiesel, biocrude and biogas generated per year in tonne/year; HHV*_i_* is the higher heating value of the *i*th useful product in MJ/tonne; *p_i_* is the unit market price of the *i*th useful product in $US.

The economic assessment metric of Equation (9) is based on the energy content of the useful streams thus emphasising the product streams of biocrude, biodiesel and biogas that are typically energy dense. This economic assessment metric therefore erroneously considers the biochar product stream as less valuable since it presents a low HHV of 4.58 MJ/tonne [7,34]. Thus, since Equation (9) does not consider the value of biochar as a viable soil additive, it may present a distorted view of the performance of the biorefinery system. The economic assessment metric of Equation (10) emphases the market prices of biocrude, biodiesel, biogas and biochar. Unfortunately, at the time of preparing this manuscript, there is no data available in the literature highlighting the market price of biocrude and biochar products, thus limiting the applicability of Equation (10). The economic assessment metric of Equation (11) considers all product streams as equally valuable, since the equation defines the production cost per unit mass of useful products, *C_m_*, with the mass, *m_p,i_*, of each useful product *i* employed as a unifying quantitative input in economic performance estimation.

Therefore, due to the limitations of Equations (9) and (10) discussed above, the present study will employ production cost per unit mass of the useful products as a sufficient economic assessment metric for economic performance assessment. This is because the production cost per unit mass of useful products, *C_m_*, does not consider any of the products (such as biochar) less valuable than others (biodiesel, biocrude and biogas) as in Equation (9) and also does not require the knowledge of the market prices of the products, as in Equation (10). Since Equation (11) employs the mass of each product stream as the unifying property of the biorefinery system in the absence of the highlighted limitations other economic performance assessment metrics discussed above, the production cost per unit mass of useful product will constitute a satisfactory indicator of the economic performance of the meat processing waste biorefinery system. The equations employed in unit product cost estimation previously reported in Reference [5] have therefore been employed.

In Equation (11), therefore, *C_T_* is calculated as follows [46],

(12) $$C_{T,j}=C_{AECC,j}+C_{AOC,j}$$

where *C_AECC,j_* represents the annual equivalent capital cost in $US in year *j* and *C_AOC,j_* represents the annual operating cost in $US in year *j*.

In Equation (12), the annual equivalent capital cost in year *j*, *C_AECC,j_*, can be estimated using the following equation [46],

(13) $$C_{AECC,j}=I_{t,j}\times \left[\frac{{\left(1+i\right)}^{n}\times i}{{\left(1+i\right)}^{n}-1}\right]$$

where, *i* represents the interest rate, specified as 10%, *n* represents the plant lifespan, assumed to be 10 years and *I_t,j_* represents the total investment cost in $US in year *j*, which is estimated as follows,

(14) $$I_{t,j}=I_{M,j}+I_{HEN,j}$$

In this Equation (14), *I_M,j_* represents the investment cost of major equipment in $US in year *j* and *I_HEN,j_* represents the investment cost of the heat exchanger network (after heat integration) in $US in year *j*. The investment cost of major equipment *I_M_* can be evaluated by the following equations [46,47],

(15) $$I_{M,j}=1.81\times E_{ISBL,j}$$

(16) $$E_{ISBL,j}=f_{L}\sum_{i}^{n}Cost_{i,j}$$

where, *E_ISBL_* represents the inside battery limit equipment cost in $US per year *j*, *f_L_* represents the Lang factor, given as 3.60 for mixed fluid-solid processing plants [45,46] and *Cost_i_* represents the equipment purchase cost for the *i*th equipment in $US in year *j*.

To calculate the investment cost of the heat exchanger network (after heat integration), the default costing methodology in ASPEN energy analyser^®^ V.10 is used to estimate the investment cost of the heat exchanger network (HEN) in $US for year 2016, as follows,

(17) $$I_{HEN,2016}=10,000+800N{\left(\frac{A}{N}\right)}^{0.8}$$

where *A* represents the area of the heat exchanger network in m^2^ and *N* represents the number of shell and tube heat exchangers; *I*_*HEN*,2016_ represents the cost of the HEN in year 2016 and is applied in Equation (14) above.

Equipment costing and sizing have been calculated using the ASPEN process economic analyser (APEA). Given that the APEA database are based on equipment cost data from 2016 (ASPEN technology Inc., personal communication, 1 August 2017) the chemical engineering plant cost index (CEPCI) is utilised in estimating the current capital plant cost for the year, 2018 (data for 2019 not available at this time), as follows [48],

(18) $$I_{t{,}_{2018}}=I_{t{,}_{2016}}\left(\frac{{\mathrm{CEPCI}}_{2018}}{{\mathrm{CEPCI}}_{2016}}\right)$$

In Equation (18), *I*_*t*,2016_ is the total investment cost, calculated based on equipment purchase costs in year 2016 (Equation (14)). The values for CEPCI_2018_ and CEPCI_2016_ were reported on the chemengonline website as 576.4 (as at 2018) and 541.7 respectively.

The purchase cost of the mechanical stirrers is not estimated by the ASPEN process economic analyser since ASPEN plus assumes complete mixing as discussed above. Therefore, the purchase cost of the mechanical stirrers is introduced to the equipment purchase cost. Assuming the mechanical stirrers utilised in each reactor vessel is a propeller type, the purchase cost of the mechanical stirrers is estimated as follows [48,49],

(19) $$Cost_{s,2016}=4866.924+2173.138S^{0.8}$$

where *Cost*_*s*,2016_ is the cost of the stirrer in $US, in 2016 and *S* is the electrical power requirement of the stirrer in kW. This calculated cost is employed in Equation (16).

The annual operating cost in year 2018, *C_AOC,_*_2018_, in Equation (12), refers to the cost associated with utilities such as energy, labour, repairs, maintenance and raw materials consumed by the biorefinery per year can be estimated as follows [48,50],

(20) $$C_{AOC,2018}=L_{c}+C_{c}+D_{c}+R_{m}+E_{c}+V_{c}$$

where *L_c_* represents the labour cost in $US, *C_c_* represents the chemical cost in $US, *D_c_* represents the depreciation cost in $US, *R_m_* represents the repair and maintenance cost in $US, *E_c_* represents the energy cost in $US and *V_c_* represents the overhead cost in $US.

All operating costs have been therefore also been evaluated for the year, 2018. These parameters are estimated as follows [46,47,48,49,50],

(21) $$L_{c}=\left[\left(15\times l_{f}\right)+\left(3\times l_{s}\right)\right]$$

(22) $$C_{c}=t\sum_{i}^{n}u_{c,i}\times {\dot{m}}_{c,i}$$

(23) $$D_{c}=\frac{I_{t,2018}}{n}$$

(24) $$R_{m}=0.06\times I_{t,2018}$$

(25) $$E_{c}=\left[\left(u_{h}\times h\right)+\left(u_{e}\times e\right)\right]$$

(26) $$V_{c}=0.05\left(D_{c}+L_{c}+E_{c}\right)$$

In Equations (21)–(26), *l_f_* represents the labour cost per year for each plant worker and specified as $US 36,672/year [51], *l_s_* represents the labour cost per year for each supervisor specified as $US 56,000/year [52], the constant values 15 and 3 refer to the assumed number of plant workers and supervisors required onsite; *u_c,i_* is the unit cost of the *i*th chemical in $US/kg (from a commercial website-alibaba.com, assessed on the 24th of February 2018), ${\dot{m}}_{c,i}$ is the mass feed rate of the *i*th chemical in kg/year; *t* is the time in years; *u_h_* is the unit heating cost specified as $US 2.48 × 10^−6^ per kJ [53], *h* is the total heat energy per year in kJ, *u_h_* is the unit electrical energy cost specified as $US 0.0681 kW^−1^ h^−1^ [54] and *e* is the total electrical energy per year in kW h.

As discussed in [5], it is assumed that fresh batches of the resin are introduced every three months. It is also assumed that fresh batches of the solid 12-tungstophosphoric acid catalyst, employed during DFA esterification reactions, are required every three months. The solid 12-tungstophosphoric acid catalyst has also assumed to be localised within the reactive distillation column to greatly simplify the simulation study.

### 3.3. Mass Feed Rate of the Processing Waste Streams

Processing variables such as the conditions of temperature and pressure and physiochemical properties of the waste streams may influence the performance of the biorefinery system. However, a comprehensive consideration of the biorefinery design challenges suggests that the mass feed rate of the meat processing waste streams of stockyard (SY) waste and DAF sludge may constitute very important parameters that may vary significantly and also influence the viability of the biorefinery system. This is because the mass feed rate of DAF sludge influences not only the biodiesel yield from the hydrolysis and esterification of the DSL and DFA respectively but also influences the mass feed rate of the WHDS residue. The mass feed rate of the WHDS residue in turn, influences the mass feed rate of SY waste, with the mass feed rate of the WHDS and SY waste mixture influencing the biogas potential from the anaerobic co-digestion process. The mass feed rate of the digestate by-product from the anaerobic co-digestion influences the biocrude, biochar, gas and post-HTL water yield from the HTL process. Clearly the mass feed rates of the waste streams (SY waste and DAF sludge) will therefore significantly influence the overall environmental performance and economic performance of the biorefinery system.

In this study therefore, the variation in the environmental performance, in terms of the NER value and the variation in the economic performance, in terms of unit production cost of the useful products, *C_m_*, is assessed. To simplify future reference to the total meat processing waste stream, the total meat processing waste stream of DAF sludge and SY waste will be represented using total meat processing waste stream (TMPS) as an abbreviation in subsequent texts. The dependence of the NER and the dependence of the unit production cost *C_m_* on the mass feed rate TMPS, is assessed for different mass feed rates, ranging from 50% to 150% of the base case. For clarity the base case is defined as the biorefinery system that can process the mass feed rates of the DAF sludge (41.7 tonnes/h), SY waste (dependent on the mass feed rate of DAF sludge based on the discussions above) and TMPS (equal to sum of mass feed rate of DAF sludge and mass feed rate of SY waste). All the analysis steps specified in Section 3 above have been carried out for the different scenarios of the processing waste mass feed rates.

### 3.4. Optimization of Biorefinery System

It is important to determine the optimum mass feed rate of TMPS in order to obtain enhanced environmental performance and enhanced economic performance of the biorefinery system. Since the economic performance and the environmental performance may be in competition with each other, biorefinery optimization is achieved when process conditions that enable the best compromise between the economic performance and the environmental performance are determined. To determine the mass feed rate of TMPS for such a compromise, a multiple objective optimization of the objective functions of environmental performance and the economic performance has been undertaken in this study. Multi-objective optimization was achieved using the numerical optimization algorithm in the JMP software (Version 14.0.0., SAS Inc., Cary, NC, USA). This optimization algorithm applies the extensively employed numerical desirability function in converting the objective functions of economic and environmental performances into a single objective function for easy optimization as discussed in previous studies [55,56,57]. Extensive discussions relating to this optimization methodology are also presented elsewhere in References [58,59,60,61]. Therefore, using the JMP software, the value of the independent variable (mass feed rate of TMPS stream) that best provides a trade-off between competing responses (environmental performance and economic performance) therefore has been determined.

## 4. Results and Discussions

### 4.1. Description of the Modelled Process

**Figure 2:**
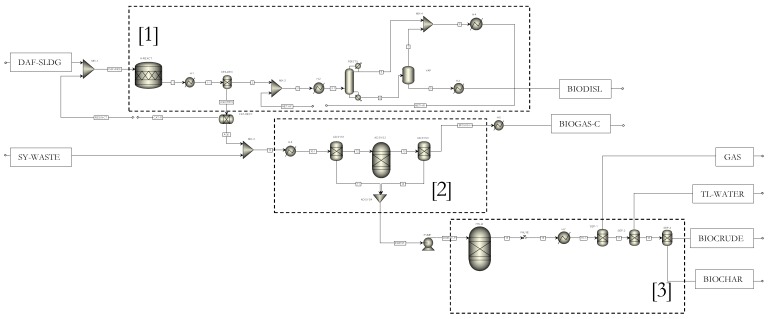
The simplified process flow sheet for the simulated meat processing waste biorefinery for biodiesel production process, anaerobic digestion process and hydrothermal liquefaction process. DAF-SLDG: dissolved air flotation sludge, SY-WASTE: stockyard waste, TL-WATER: hydrothermal liquefaction water, RESIN-CT: Resin catalyst, DAF+RES: Dissolved air flotation sludge plus catalyst, CAT-R: Catalyst recovered, DFA-DEC: Fatty acid separation unit, CAT-RECY: Catalyst recycle; AQU+RES; Aqueous phase products plus resin; METH-F: Methanol feed; METH-R: Methanol recovered, CAT-R: Catalyst recovered, AQU: Aqueous phase products, BIODISL: Biodiesel; RDISTIL, Reactive distillation column; VAP, Vaporiser; H-1 to H-7: Heat exchangers 1 to 7 respectively, SEP-1, SEP-2 and SEP-3: Employed in separation operations 1, 2 and 3 respectively, DIGEST: Digestate, DIGEST-P: Pressurised digestate, AD-SYS1, AD-SYS2, AD-SYS3 and AD-SYS4: Anaerobic digestion sub-systems of 1, 2, 3 and 4 respectively and BIOGAS-C (-H): Biogas at 25 °C (37 °C).

The modelled flow chart of the biorefinery system is presented in Figure 2. In this figure, dashed blocks 1, 2 and 3 represent the major biomass conversion processes of the biorefinery system: integrated in-situ hydrolysis and esterification process, anaerobic co-digestion process and hydrothermal liquefaction process responsible for the major products of biodiesel, biogas and biocrude and biochar respectively. The detailed individual operation units and the mass streams of the biorefinery system are also shown in Figure 2. The modelled results of the mass flow rates, temperatures, pressures, mass fractions of the streams are listed in Table 1 in that table together with the assigned process input data of the mass flow rate, temperature, pressure of the feedstock streams.

#### 4.1.1. The integrated In-Situ Hydrolysis and Esterification Process

As shown in the dashed block 1 in Figure 2 and discussed in Reference [5], the inlet feed stream, wet DAF sludge (stream DAF-SLDG) containing 92 wt.% moisture content (wet basis) at a mass flow rate of 1000 t/day (41667 kg/h), temperature of 25 °C and pressure of 1 atm and the resin-catalyst stream (RESIN-CT) at a mass flow rate of 3840 kg/h, temperature of 25 °C and pressure of 1 atm, are initially mixed (MIX-1) and the mixture is fed to the in-situ lipid hydrolysis reactor (H-REACT).

The in-situ hydrolysis reaction temperature is specified as 92.5 °C and the reaction pressure specified as 1 atm as determined in [3]. Cooling of the hydrolysed product (stream 1) to 25 °C is achieved using a heat exchanger (H-1) as the cooling process is necessary to enhance the separation of non-polar DAF fatty acids from the hydrolysed mixture of the polar aqueous phase and solid phase (AQ + RES) as discussed in [6]. For simplicity, it is assumed that, 99 wt.% of DAF fatty acids (DFA) is recovered in the DFA separation process and a 100 wt.% of the resin catalyst is recovered from the catalyst recovery units. Methanol (stream METH-F) is then mixed in a mixer (MIX-2) with the recovered DFA (stream 2), with a molar ratio of methanol to fatty acid of 40 to 1. Prior to the esterification reaction in the reactive distillation column (RDISTIL), the methanol-fatty acid mixture (stream 3) is preheated to 70 °C at a pressure of 1 atm using a heat exchanger (H-2) to reduce the reboiler duty of the reactive distillation column. The esterification reaction between methanol and DAF sludge fatty acid is then undertaken to produce DAF sludge methyl ester (DSME) and also a small mass of water (reaction Equation (2)) under the action of solid 12-tungstophosphoric acid catalyst which is assumed to be localised on the trays in the reactive distillation column.

The utilisation of reactive distillation column (RDISTIL) is consistent with researches that considered the one step esterification and product separation operation undertaken in the reactive distillation column as a highly efficient technological intensification strategy that reduces the net energy requirement of biodiesel production processes [62,63]. After the esterification reaction and the separation of the DSME from the unreacted methanol and the water produced in the reactive distillation column (RDISTIL), a further purification process of the DSME stream is required, because the DSME stream (stream 4) contains unreacted methanol and water with a mass fraction (*x* = 0.785) of DSME, as shown in Table 1. Purification of stream 4 is achieved via a vaporisation operation. The vaporisation operation (VAP) is undertaken at a high temperature of 150 °C and under a pressure of 1 atm. The vapour (stream 7) generated from the vaporisation process is then mixed with the distillate (stream 5) in a mixer (MIX-4). The mixture stream (stream 8), containing 98.6 wt.% (mass fraction) of methanol as shown in Table 1, is then cooled to its liquid phase at 25 °C using a heat exchanger (H-4). The purified biodiesel product (stream 6) is also cooled to 25 °C using a heat exchanger (H-3). The cooled and purified biodiesel product (BIODISL) is shown to contain 98.9 wt.% (mass fraction) of fatty acid methyl ester (FAME) and thus satisfies the minimum required FAME content for biodiesel, specified as 96.5 wt.% according to EN 14214 European standards [64].

#### 4.1.2. The Anaerobic Co-Digestion Process

As shown in the dashed block 2 in Figure 2, the integrated in-situ hydrolysis and esterification process results in the generation of a wet hydrolysed DAF sludge (WHDS) residue stream (AQU). This WHDS stream (AQU) is used as a co-anaerobic digestion substrate together with the stockyard waste stream (SY-WASTE) to generate biogas. The mass flow rates, temperatures and pressures of the WHDS stream (AQU) and the stockyard waste stream (SY-WASTE) are listed in Table 1. Table 1 shows that the stockyard waste stream (SY-WASTE) is supplied to the anaerobic co-digestion process such that its mass feed rate is 1.029 times the mass feed rate of the WHDS stream (AQU). Table 1 also shows that the mass feed rate of the stockyard waste stream (SY-WASTE) is 1.019 times the mass feed rate of the DAF sludge (DAF-SLDG). This relation between the mass feed rate of stockyard waste and DAF sludge will be a valuable input in discussions presented in subsequent Sections. Figure 2 shows that the AQU and SY-WASTE streams are mixed using a mixer (MIX-3) and then the mixed stream (stream 10) is heated in a heat exchanger H-6 to the mesophilic temperature condition of 37 °C and the moisture content is adjusted to 7.26 times the mass of total solids [6] of the mixed stream (stream 10-1) in the process denoted by AD-SYS1. The AD process is carried out in the digester represented by AD-SYS2, which is modelled using the method of Gibbs free energy minimisation discussed above. The biogas separation from the wet digestate is modelled as a separation process in a separator represented by AD-SYS3. The mixing process of the unreacted substrate from AD-SYS3 and water from AD-SYS1 is carried in the mixer denoted by AD-SYS4. The generated biogas stream (BIOGAS-H) is cooled in a heat exchanger (H-5) to the temperature 25 °C of the biogas stream (BIOGAS-C).

#### 4.1.3. The Hydrothermal Liquefaction Process

As shown in the dashed block 3 in Figure 2, the digestate (DIGEST) from the anaerobic co-digestion process is pressurized to 12 MPa (or 118.431 atm) using a pump (PUMP) and fed to the HTL reactor (HTL-R). The HTL reaction is carried out at a temperature of 257 °C and pressure of 12 MPa (or 118.431 atm) [7,33]. At the conclusion of the HTL process the high pressure product stream (stream 15) is depressurized (VALVE) to a low pressure of 1 atm (stream 16) and subsequently cooled to 25 °C using a heat exchanger (H-6) at atmospheric pressure. According to Jones et al. [65], a large-scale separation process of HTL products is achieved easily by exploring the differences in the immiscibility and surface properties of the insoluble biochar solids (BIOCHAR), hydrophobic biocrude (BIOCRUDE) and the polar post-HTL water (TL-WATER). Separation of the biocrude, post-HTL water, biochar and gases is therefore simulated using simple separation models in ASPEN plus, as SEP-1, SEP-2 and SEP-3 in Figure 2, respectively.

### 4.2. Energetic Analysis and Environmental Performance Results

The thermal data of source and target temperatures, heating duties and cooling duties of the hot and cold streams in the biorefinery system, have been extracted from the simulation results and listed in Table 2. Table 2 shows that prior to heat integration, the cooling duties and heating duties associated with the hot streams and the cold streams in the biorefinery system are 28872.8 kW and 25621.0 kW respectively. This indicates that there are opportunities for heat recovery.

Considering the duties of the hot and cold streams presented in Table 2, the hydrothermal liquefaction biomass conversion process constitutes the major energy demanding operation with the HTL reactor (HTL-R_heat) being responsible for the highest heating utility requirement of 20,551.6 kW and having the highest target temperature of 257 °C. This high heating duty reflects the large heat energy required to raise the temperature of the inlet stream (DIGEST-P, Figure 2) from 38.8 °C to the high temperature of 257 °C and maintain the high temperature the hydrothermal liquefaction reacting mixture (electrical power requirement of the pump for pressure of 12 MPa is discussed below). From Table 2, it is seen that the cooling duty 24,071.9 kW, required for cooling the stream at the exit of the HTL reactor from 98.3 °C of stream 16 to 25 °C of stream 17 constitutes the largest cooling duty in the overall biorefinery system. The thermal data listed in Table 2 has been utilised to identify opportunities for heat recovery to reduce the heat duty requirement of the biorefinery system, as discussed in Section 3.1 above.

**Figure 3:**
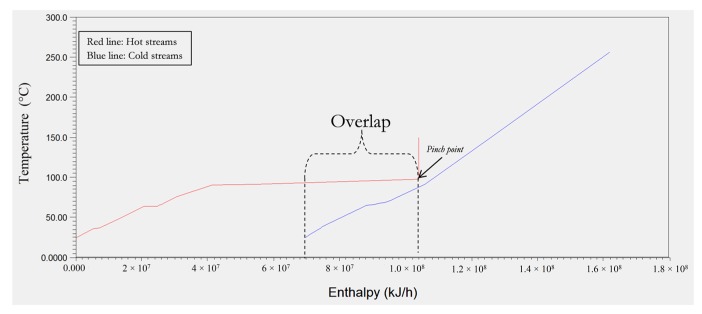
Composite curves for minimum driving temperature of 10 °C for the biorefinery process as generated by ASPEN energy analyser^®^.

Using the ASPEN energy analyser^®^ V.10, the composite curves, which represent the total heating and the total cooling requirements of the biorefinery process in a cumulative manner on a temperature–enthalpy diagram, have been generated and are shown in Figure 3, based a counter-current heat exchanger network and a temperature difference of 10 °C. The plot employs the classic heat integration method which is extensively described in chemical engineering textbooks and journals [38,66,67]. It has been determined that employing 18 heat exchangers, the cooling utility and the heating utility associated with the hot and the cold streams (Table 2) for the biorefinery process can be reduced from 28,872.8 kW (or 103,942.1 MJ/h) and 25,621.0 kW (or 92,235.6 MJ/h) to 19,370 kW (or 69,732 MJ/h) and 16,120 kW (or 58,032 MJ/h) respectively. The pinch point temperature was also determined to be 98.3 °C.

The residual cooling and the heating requirements may be satisfied using cooling water and steam (generated using natural gas) as external utilities. In addition to duties associated with the hot and cold streams, additional auxiliary heating and cooling duties due to mixing heat of isothermal and isobaric separation operations were also calculated [68,69]. The total auxiliary heating duty and cooling duty in the biorefinery system were determined in ASPEN as 1790 kW (or 64.44 MJ/h) and 447.8 kW (or 1612 MJ/h) respectively. The electrical duty for the high pressure HTL pump has been calculated to be 386 kW (or 1389.6 MJ/h). Furthermore, as discussed in above, the combined electrical power required by the mechanical stirrers employed in the in-situ hydrolysis reactor (H-REACT), anaerobic digestion reactor (AD) and hydrothermal liquefaction reactor (HTL-R) has also estimated to be 103.1 kW (or 371.16 MJ/h). The values of the external energy duties required by the biorefinery system discussed above are summarised in Table 3 below.

In this study, only the yields of the energy dense product streams of biocrude, biodiesel and biogas of 722.5, 425.6 and 1564.3 kg/h and their associated HHVs of 36,700, 39,800 and 20,900 kJ/kg respectively are considered in calculating the total thermal energy generation potential of the biorefinery system. Applying Equation (8) above, the NER of the overall biorefinery system has been determined to be 1.010 for the case of electricity generation from fossil sources (case A) and 1.063 for the case of electricity generation from renewable sources (case B). The NER results show that for the biorefinery system in both case A and case B (NER_A_ and NER_B_ are greater than 1), energy recovery via the employment of the proposed biorefinery cannot result in significant positive environmental outcomes since both NER values are not substantially greater than 1.

### 4.3. Economic Assessment Results

The purchase cost of the major equipment has been estimated using the ASPEN process economic analyser^®^ V.10 and is presented in Table 4. Table 4 shows that based on the purchase cost estimate of the equipment used in the biorefinery system, the cost of the hydrothermal liquefaction reactor (HTL-R) constitutes the highest purchase cost requirement in the biorefinery system. This high purchase cost estimate is a reflection of not only the large capacity of the reactor but also the need for a high-temperature (257 °C) and a high-pressure (12 MPa) reactor vessel. In other words the introduction of the hydrothermal liquefaction process has both energetic and economic unfavourable consequences on the biorefinery process. Overall the annualised capital cost for the overall process is estimated to be about $US 1.5 million. The operating cost components were estimated and presented in Table 5. Table 5 shows that manpower as represented by the labour cost ($kUS 718.08) is the major cost contributor to the total operating cost.

Combining the operating cost per year and the annualised capital cost estimated and listed in Table 4 and Table 5 respectively, the total cost incurred per year is estimated to be $kUS 4077.2. Based therefore on the estimated yields of biochar, biocrude, biodiesel and biogas of 4894.5, 722.5, 425.6 and 1564.3 kg/h respectively, the unit cost of producing useful products has been estimated using Equation (11), to be $US 74.4 per tonnes of total mass of the useful products.

### 4.4. Dependence of Environmental and Economic Performance on Mass Feed Rate of Waste Streams

The effect of varying the mass feed rates of the waste streams on the economic and environmental performances have been initially investigated and the economic data and energetic data generated and presented in Tables S1 and S2 respectively in the supplementary data document. Tables S1 and S2 show that a constant ratio of the mass feed rate of SY waste to the mass feed rate of DAF sludge of 1.019 is maintained. This implies that if the mass feed of the TMPS is known, then the mass feed rate of the DAF sludge can be determined as follows,

(27) $$\text{Mass feed rate of DAF sludge}=\frac{\text{Mass feed rate of TMPS}}{2.019}$$

**Figure 4:**
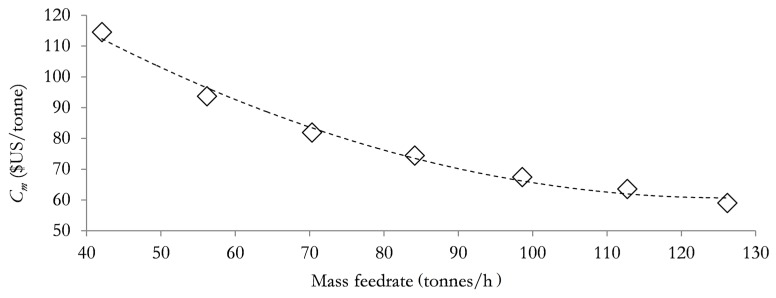
Dependence of *C_m_* on the mass feed rate of the meat processing waste streams.

**Figure 5:**
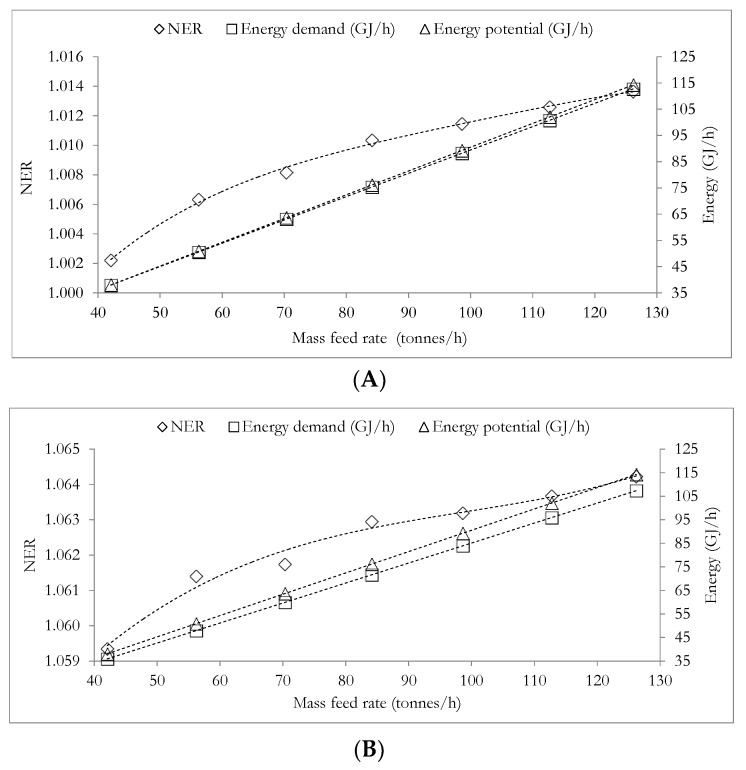
Dependence of NER, energy demand and energy potential on the mass feed rate of the total meat processing waste stream. (**A**) for case when electrical energy is generated from fossil sources and (**B**) for case when electrical energy is generated from renewable energy sources. Slight numerical fluctuations are responsible for the lack of perfect smoothness in NER versus the total meat processing waste stream (**B**).

The relation presented in Equation (27) constitutes an important relation to be employed in subsequent subsections. Figure 4 and Figure 5A,B illustrate the dependence of the unit production cost and NER, on the mass feed rate of the total meat processing waste streams (TMPS). The data employed in plotting Figure 4 and Figure 5A,B are presented in Tables S1 and S2 respectively in the supplementary data document.

Figure 4 and Figure 5A,B highlight the plot of unit production cost (*C_m_*) versus the mass feed rate of the meat processing waste streams and the plot of NER versus the mass feed rate of the meat processing waste streams for case A (Figure 5A) and case B (Figure 5B) respectively. Figure 4 and Figure 5A,B show that as the mass feed rate of meat processing waste streams of DAF sludge, SY waste and mass feed rate of TPMS increases, the unit production cost reduces and the NER value increases. This observation implies that both the environment performance and economic performance of the biorefinery system will benefit from larger biorefinery processing capacities, with larger mass feed rates of meat processing waste streams leading to reduced processing cost and improved NER values in both cases A and B (discussed in Section 3.1 above). Figure 4 and Figure 5A,B also suggest that changes in the DAF feed rate will present a greater effect on the unit production cost than on the NER value since the unit production cost reduces by as much as 52% while the NER value increases by only about 1.14% (case A) and 0.46% (case B) as the mass feed rate of meat processing waste streams increases from 50% to 150% of the base case mass feed rates. It is however not certain that the above situation of simultaneously favourable environmental performance will still be satisfactory if the mass feed rates DAF sludge, SY waste and TMPS are greater than 150% of their base case mass feed rates. It is therefore necessary to be further determined. Since mass feed rates DAF sludge and the SY waste can be determined from the mass feed rate of the TMPS as discussed in Section 4.1 and using Equation (27) above, the effect of increasing the mass feed rates of only the TMPS to greater than 150% of the base case mass feed rate of 84.2 tonnes/h, on the NER and the unit production cost of the biorefinery system has been assessed.

**Figure 6:**
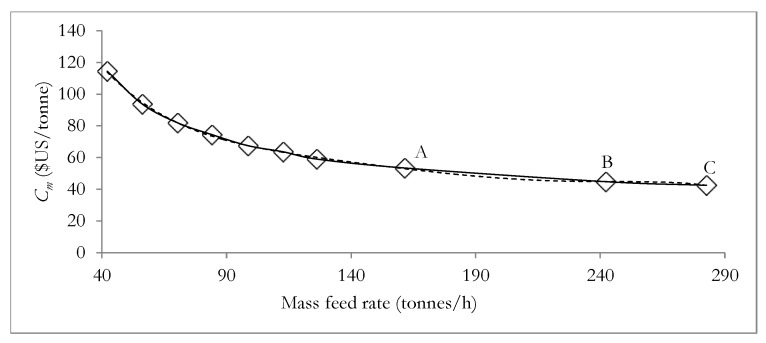
Variation of the unit production cost with the mass feed rates. Points A, B and C represent mass feed rates of TMPS that are 192%, 288% and 336% (all greater than 150%) of the base case TMPS mass feed rate of 84.2 tonnes/h.

**Figure 7:**
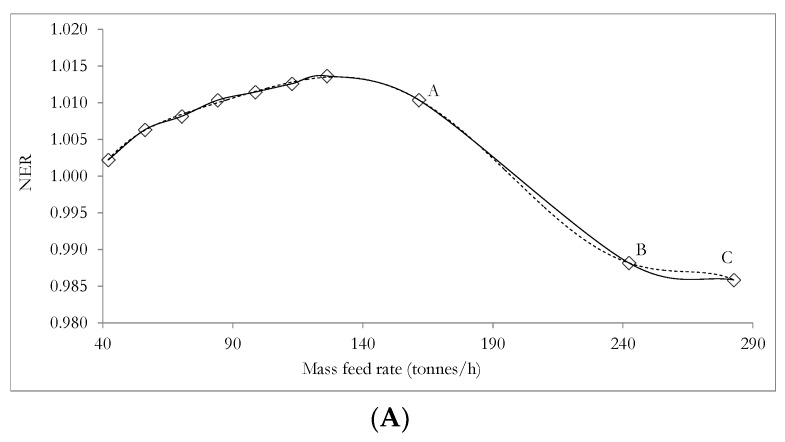

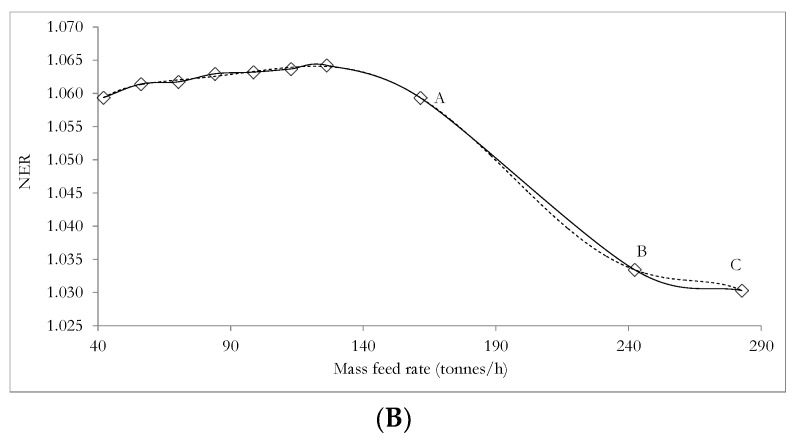
Variation of the NER with the mass feed rates. Points A, B and C represent mass feed rates of TMPS that are 192%, 288% and 336% (all greater than 150%) of the base case TMPS mass feed rate of 84.2 tonnes/h. (**A**): Case A and (**B**): case B.

The NER and the unit production cost of the biorefinery system have therefore been further investigated for several mass feed rates of TMPS of 161.5 tonnes/h (A), 242.3 tonnes/h (B) and 282.7 tonnes/h (C) which are all greater than 150% of the base case mass feed rate of 126.2 tonnes/h. The modelled results from ASPEN plus are presented in Tables S3 and S4 in the supplementary data document and the data used in generating plots showing the dependence of the unit production cost, *C_m_* and NER on the mass feed rate of TMPS as shown in Figure 6 and Figure 7A,B respectively. Figure 6 and Figure 7A,B show that when the mass feed rates of the TMPS are 161.5 tonnes/h, 242 tonnes/h and 282.7 tonnes/h, corresponding to points A, B and C on the respective plots, the same trend as shown in Figure 6 is observed with the unit production cost, *C_m_*, reduced to $US 53.39/tonne, $US 44.76/tonne and then $US 42.48/tonne as the mass feed rate of TMPS increases further from 161.5 tonnes to 242 tonnes and then 282.7 tonnes respectively. However Figure 7A,B show different trends from the trends shown in Figure 7A,B for NER is shown, with Figure 7A showing that the NER reduces from 1.010 to 0.988 and then 0.986 as the mass feed rates of the TMPS further increases from 161.5 to 242 and then 282.7 tonnes. Figure 7B also shows that the NER reduces from 1.059 to 1.033 and then 1.030 as the mass feed rates of the TMPS further increases from 161.5 to 242 and then 282.7 tonnes.

Figure 6 shows that a larger mass feed rate of the waste streams will always lead to a reduced unit production cost, with the production cost reducing asymptotically towards a possible constant value. In other words in the absence of the environmental performance consideration, increasing the mass feed rate of the waste streams processed by the biorefinery system will constitute the logical approach to enhance the economic performance. Figure 7A,B however show that with an increase in the mass feed rate of the waste streams the NER increase initially, peak and then begin to deteriorate.

This indicates that at a high mass feed rate of the TMPS the energy cost necessary for processing of the waste streams to useful products will exceed the energy potential of the energy dense product streams of biodiesel, biocrude and biogas leading to poorer environmental performance (i.e., lower NER). To determine the most acceptable feed rate of the TMPS, for the best compromise performances for NER and unit production cost (Section 4.1), it is necessary to establish the relations between the mass feed rates of TMPS and the NER and the relation between the mass feed rates of TMPS and the unit production cost.

A least square regression line fitting method [70] in JMP^®^ statistical software version 10.0.0 (SAS Institute Inc., Cary, NC, USA) has been employed to develop relevant fitted relations. For the mass feed rate of TMPS ranging from 42 tonne/h to 282.7 tonnes/h, the following two fitted relations for the unit production cost (*C_m_*) and the NER for case A (NER_A_) and case B (NER_B_) have been established respectively, as follows,

(28) $$\begin{array}{ll} C_{m} & =-9.68\times 10^{-10}{\left(m\right)}^{5}+8.72\times 10^{-7}{\left(m\right)}^{4}-3.05\times 10^{-4}{\left(m\right)}^{3}+5.26\times 10^{-2}{\left(m\right)}^{2}\\ & -4.70\left(m\right)+238.89 \end{array}$$

and

(29) $$\begin{array}{ll} {\mathrm{NER}}_{A} & =-1.03\times 10^{-14}{\left(m\right)}^{6}+9.8\times 10^{-12}{\left(m\right)}^{5}-3.59\times 10^{-9}{\left(m\right)}^{4}+6.4\times 10^{-7}{\left(m\right)}^{3}\\ & -6.06\times 10^{-5}{\left(m\right)}^{2}+3.01\times 10^{-3}\left(m\right)+0.9449 \end{array}$$

(30) $$\begin{array}{ll} {\mathrm{NER}}_{B} & =-1.1\times 10^{-14}{\left(m\right)}^{6}+1\times 10^{-11}{\left(m\right)}^{5}-3.64\times 10^{-9}{\left(m\right)}^{4}+6.37\times 10^{-7}{\left(m\right)}^{3}\\ & -5.77\times 10^{-5}{\left(m\right)}^{2}+2.64\times 10^{-3}\left(m\right)+1.013 \end{array}$$

In the three equations above, *m* is the mass feed rate of TMPS in tonnes/h and ranging from 42 to 282.7 tonnes/h. The coefficient of determination (*R*^2^) for Equations (28)–(30) are 0.9992, 0.9997 and 0.9998, respectively. The three relations are shown in Figure 6 and Figure 7A,B, as the dashed curves for unit production cost (*C_m_*) versus mass feed rate plot and NER versus mass feed rate plot respectively.

Utilising the fitted relations presented in Equations (28)–(30) above and the JMP software for desirability optimisation as discussed in Section 4.1 above, the mass flow rate of TMPS, that will provide a best compromise trade-off between the competing NER and the unit production cost, *C_m_*, has been determined and presented in Table 6. Table 6 also shows that for case B, the mass feed rate of TMPS that will enable the best compromise between competing NER and unit production cost, *C_m_* is 140.8 tonnes/h with the associated NER and unit production cost, *C_m_* of 1.063 and $US 57.0 per tonne respectively. Employing Equation (27) above, in both cases, A and B, the mass feed rate of DAF sludge can be estimated to be 71.62 and 69.74 tonnes/h respectively. Given that the ratio of the mass feed rate of SY waste to the mass feed rate of DAF sludge is maintained at 1.019 as determined above (Table 1), the mass feed rate of SY waste in cases A and B can be estimated to be 72.98 and 71.07 tonnes/h respectively.

The result clearly shows that under the optimal mass feed rate conditions of TMPS, cases of A and B will have close unit production costs of $US 56.2 per tonne and $US 57.0 per tonne respectively. The lower unit production costs of $US 56.2 per tonne calculated in case A, compared to the unit production costs of $US 57.0 per tonne in case B can be explained by economies of scale since production cost was shown to reduce as the mass of the feed rate of the TMPS increased (Figure 6 above). Case A and case B have also been initially shown to constitute environmentally sustainable processes since their NERs are 1.013 and 1.063 respectively and both NERs are greater than 1.

This preliminary assessment also suggests that assuming the meat processing waste streams of SY waste and DAF sludge are readily available on site, such that the transportation cost is negligible, a single large capacity biorefinery plant (TMPS of 144.6 tonnes/h, case A and 140.8 tonnes/h, case B) to satisfy the resource recovery needs of a particular region will be preferred to the installation of several smaller capacity biorefinery plants in that region, from a perspective of both environmental performance and economic performance. However, in the absence of sufficient meat processing waste streams of SY waste and DAF sludge in a particular region, cost escalation due to the transportation waste streams over long distances may limit the long term viability of such a large capacity biorefinery plant. This is because such cost escalations may increase the unit production cost well beyond the costing range and scope considered in this study.

### 4.5. Unit Production Cost Comparison with Data Obtained from Peer-Reviewed Literature

To provide a context for the assessment of the economic performance of the optimised biorefinery system, unit production costs of valuable products of biochar, biocrude, biogas and biodiesel have been compared to unit production costs of the respective products. Employing the determined mass flow rate of TMPS of 144.6 tonnes/h (DAF: 71.62 tonnes/h, SY: 72.98 tonnes/h) and 140.8 tonnes/h (DAF: 69.74 tonnes/h, SY: 71.07 tonnes/h) for case A and case B respectively, the mass (tonnes) of the valuable products produced per h can be calculated in ASPEN plus by undertaking the same simulation steps highlighted in Section 3 and Section 4 above. The mass of the useful products from the biorefinery in case A and case B are therefore summarised in Table 7.

Table 7 shows the mass flow rate of the valuable products and annual production costs for cases A and B of the biorefinery such that the unit production costs of each of the products is calculated and the results summarised in Table 8. Table 8 shows the production costs of useful products per unit mass and per unit volume. The conversion of the unit production cost from a mass basis to a volumetric basis has been achieved by multiplying production costs per unit mass of each product by their respective densities. These densities are specified as 0.0012 kg/L [71], 0.8692 kg/L [4], 0.974 kg/L [7,34] and 0.51 kg/L (bulk density) [72] for biogas, biodiesel, biocrude and biochar respectively.

A review of the literature shows that the unit production costs of biocrude, biochar (from waste) and biodiesel are US$ 0.85/L [73], US$ 0.26/L [74] and US$ 1.124/L (mean) [75,76,77] respectively. Since the mean production cost for biogas is approximately €14 per GJ of biogas heat content [78], the production cost of biogas per L can be estimated from the knowledge of its HHV and density of 20.9 MJ/kg [6] and 0.00115 kg/L [71] respectively. The mean unit production cost of biogas is calculated to be €0.00025/L which is equivalent to US$ 0.00029/L, based on the prevailing currency exchange rate of €1 to US$ 1.17 (assessed on the 14th of September 2018 from www.currency-calc.com).

Comparing the literature on the obtained unit production costs and the unit production costs obtained from the present study for products of biodiesel, biocrude and biochar, it is clear that the proposed biorefinery system can generate the aforementioned useful products with comparatively reduced unit production costs relative to the current unit production costs of the products found in the literature. The unit production costs of the biogas, in both cases A and B, were however estimated to be US$ 0.00031/L and US$ 0.00032/L which are comparable to the literature obtained current mean unit production cost of biogas of US$ 0.00029/L. The ability of the biorefinery to generate useful products at lower unit production costs relative to the literature obtained unit production costs of the independently produced respective products may be largely due to the generation of multiple product streams from the proposed biorefinery system. This observation is consistent with a previous study in which it was shown that the production of a single useful product may not constitute the most economically favourable approach for organic waste utilisation, with the production of multiple useful product streams from an organic waste feedstock preferred for improved economic performance [79]. It is therefore clear that the proposed meat processing waste biorefinery enables the recovery of useful products of biodiesel, biocrude and biochar from meat processing waste at reduced unit production costs.

## 5. Conclusions

This study has presented an assessment of the economic performance and environmental performance of the proposed large-scale meat processing waste biorefinery system for the recovery of valuable materials and for the production of valuable products: biocrude, biodiesel and biogas products (biofuels) and biochar (biomaterial). In this study, two cases, A and B—denoting systems in which electricity generation employs fossil energy sources and renewable energy sources respectively—have been investigated. It was demonstrated that some synergy initially exists between the economic performance and environmental performance of the biorefinery system as the mass flow rate of the meat processing waste stream increases. The model results show that an increase in the mass feed rate of the meat processing waste from 50% to 150% of the base case mass feed rate has positive effects on economic and environmental performance. The positive effect of increasing the mass flow rates of the meat processing waste stream on economic performance is shown to be greater than the positive effect on environmental performance. This is because the increase in the mass feed rate of meat processing waste stream from 50% to 150% of the base case mass feed rate of the waste stream resulted in a 1.14% and 0.46% improvement in environmental performance in case A and case B respectively while a 52% improvement in economic performance was determined. However, a further increase in the mass feed rates of the meat processing waste to 192%, 288% and 336% of the base case mass feed rate, was observed to lead to a deterioration in environmental performance in both cases A and B. The total mass feed rate of the waste stream that resulted in a satisfactory compromise between the competing environmental performance and economic performance was determined to be 144.6 and 140.8 tonnes/h in cases A and B respectively. This high value of the mass feed rate in both cases suggests that a biorefinery system with a large capacity could be preferable to smaller ones from both economic and environmental perspectives, provided of course that the waste streams are readily available in close proximity to the biorefinery system and therefore reduce the risk of transportation cost escalation. Most importantly this study was able to show that when the determined compromise mass flow rates of the meat processing waste stream in cases A and B are processed by the biorefinery system, a reduction in the unit production cost of biocrude, biodiesel (biofuels) and biochar (biomaterial) was feasible without sacrificing the need for environmental sustainability. The unit production cost of biogas (biofuel) was however shown to be comparable to the existing unit production cost of biogas, according to the literature.

## Figures and Tables

**Table 1:** The simulation results of the major streams in the process flow sheet for the proposed biorefinery system.

**Stream name**	**DAF-SLDG**	**SY-WASTE**	**1**	**1-1**	**2**	**3**	**3-1**	**4**	**5**	**6**	**7**	**8**	**10**	**10-1**	**11**	**12**
Temperature (°C)	25.0	25.0	92.5	25.0	25.0	25.0	70.0	76.7	64.7	150.0	150.0	64.9	25.0	37.0	37.0	37.0
Pressure (atm)	1	1	1	1	1	1	1	1	1	1	1	1	1	1	1	1
Mass fractions (*x*)																
DSL (*x*)	0.010	0.008	0	0	0	0	0	0	0	0	0	0	0.004	0.004	0.004	0.004
DFA (*x*)	0	0	0.009	0.009	1.00	0.175	0.175	Trace	Trace	Trace	Trace	Trace	Trace	Trace	Trace	Trace
Water (*x*)	0.920	0.833	0.842	0.842	0	0	0	0.033	0.005	0.004	0.145	0.013	0.880	0.880	0.880	0.881
Glycerol (*x*)	0	0	0.001	0.001	0	0	0	0	0	0	0	0	0.001	0.001	0.001	0.001
Methanol (*x*)	0	0	0	0	0	0.825	0.825	0.182	0.995	0.008	0.847	0.986	0	0	0	0
Ash (*x*)	0.017	0.057	0.016	0.016	0	0	0	0	0	0	0	0	0.038	0.038	0.038	0.037
Carbohydrate (*x*)	0.035	0.077	0.032	0.032	0	0	0	0	0	0	0	0	0.056	0.056	0.056	0.056
Protein (*x*)	0.017	0.025	0.016	0.016	0	0	0	0	0	0	0	0	0.021	0.021	0.021	0.021
DSME (*x*)	0	0	0	0	0	0	0	0.785	Trace	0.989	0.008	0	0	0	0	0
Resin (catalyst) (*x*)	0	0	0.084	0.084	0	0	0	0	0	0	0	0	0	0	0	0
Ammonia (*x*)	0	0	0	0	0	0	0	0	0	0	0	0	0	0	0	0
Carbon dioxide (*x*)	0	0	0	0	0	0	0	0	0	0	0	0	0	0	0	0
Biomethane (*x*)	0	0	0	0	0	0	0	0	0	0	0	0	0	0	0	0
Biocrude (*x*)	0	0	0	0	0	0	0	0	0	0	0	0	0	0	0	0
Nitrogen (*x*)	0	0	0	0	0	0	0	0	0	0	0	0	0	0	0	0
Mass Flows (kg/h)	41,666.7	42,460.7	45,506.7	45,506.7	401.0	2292.8	2292.8	537.4	1755.4	425.6	111.8	1867.2	83,726.4	83,726.4	74,610.2	9116.1
**Stream name**	**13**	**14**	**15**	**16**	**16-1**	**DIGEST**	**AQU**	**BIOCHAR**	**BIOCRUDE**	**BIODISL**	**BIOGAS-C**	**CAT-R**	**DAF+RES**	**GAS**	**TL-WATER**	
Temperature (°C)	37.0	37.0	257.0	98.3	25.0	37.0	25.0	25.0	25.0	25.0	25.0	25.0	30.2	25.0	25.0	
Pressure (atm)	1	1	118.43	1	1	1	1	1	1	1	1	1	1	1	1	
Mass fractions (*x*)																
DSL (*x*)	0	0	0	0	0	0.004	0	0	0	0	0	0	0.010	0	0	
DFA (*x*)	0	0	0	0	0	Trace	Trace	0	0	Trace	0	0	0	0	0	
Water (*x*)	0.828	1	0.909	0.909	0.909	0.891	0.929	0	0	0.004	0	0	0.843	0	1	
Glycerol (*x*)	0	0	0	0	0	0.001	0.001	0	0	0	0	0	0	0	0	
Methanol (*x*)	0	0	0	0	0	0	0	0	0	0.008	0	0	0	0	0	
Ash (*x*)	0	0	0.06	0.06	0.06	0.035	0.018	1	0	0	0	0	0.016	0	0	
Carbohydrate (*x*)	0	0	0	0	0	0.051	0.035	0	0	0	0	0	0.032	0	0	
Protein (*x*)	0	0	0	0	0	0.019	0.018	0	0	0	0	0	0.016	0	0	
DSME (*x*)	0	0	0	0	0	0	0	0	0	0.989	0	0	0	0	0	
Resin (catalyst) (*x*)	0	0	0	0	0	0	0	0	0	0	0	1	0.084	0	0	
Ammonia (*x*)	0.002	0	0	0	0	0	0	0	0	0	0.013	0	0	0	0	
Carbon dioxide (*x*)	0.111	0	0.021	0.021	0.021	0	0	0	0	0	0.647	0	0	0.930	0	
Biomethane (*x*)	0.058	0	0	0	0	0	0	0	0	0	0.340	0	0	0	0	
Biocrude (*x*)	0	0	0.009	0.009	0.009	0	0	0	1	0	0	0	0	0	0	
Nitrogen (*x*)	0	0	0.002	0.002	0.002	0	0	0	0	0	0	0	0	0.070	0	
Mass Flows (kg/h)	9116.1	7551.9	82,162.3	82,162.3	82,162.3	82,162.3	41,265.6	4894.5	722.5	425.6	1564.3	3840.0	45,506.7	1899.1	74,646.2	

**Table 2:** Hot and cold streams extracted from the simulation datasheet sheet for the biorefinery process.

Stream Description	Temperature (°C)		Duty (Enthalpy Change) (kW)
	Source	Target	
Hot streams	–	–	–
8_to_METH-R	64.9	25	113.0
BIOGAS-H_To_BIOGAS-C	37	25	7.0
1_To_1-1	92.5	25	3130.7
AD-SYS2_heat	37	36.5	441.6
RDISTIL condenser_TO_5	65.5	64.7	1076.76
16_To_17	98.3	25	24071.9
6_To_BIODISL	150	25	31.8
Sum	–	–	28872.8
Cold streams	–	–	–
10_To_10-1	25.0	37	999.3
RDISTIL reboiler_TO_4	67	78.3	580.6
HTL-R_heat	38.8	257	20551.6
VAP_heat	76.7	150	62
3_To_3-1	25	70	597.7
H-REACT_heat	30.2	92.5	2829.8
Sum	–	–	25621

**Table 3:** External energy duty requirements of the simulated biorefinery process.

Energy Demand	Value Calculated (kW)
Heating duty	
Minimum heating duty	16,120.0
Auxiliary heat duty	1790.0
Total	17,910.0
Cooling duty	
Minimum cooling duty	19,370.0
Auxiliary cooling duty	447.8
Total	19,817.8
Electrical duty	
Pumps	386.0
Stirrers	103.1
Total	489.1

**Table 4:** Purchase and investment costs for the biorefinery process (Equations (9)–(19)).

Equipment_year_	Purchase Cost ($kUS)
H-REACT_2016_	124.8
RDISTIL-condenser acc_2016_	13
RDISTIL-reflux_2016_	4.6
RDISTIL-tower_2016_	85.2
CAT-RECY_2016_	18.2
AD-SYS3_2016_	18.2
SEP-2_2016_	29.5
AD-SYS1_2016_	18.2
DFA-DEC_2016_	15.7
VAP-flash vessel_2016_	15.7
AD-SYS2_2016_	75.5
SEP-1_2016_	29.5
HTL-R_2016_	339.8
PUMP_2016_	230.6
SEP-3_2016_	15.7
Stirrers	124.5
Total purchase cost_2016_	1162.17
ISBL cost_2016_	3487.7
Investment cost (*I_M_*_,2016_)	7530.83
HEN Investment cost (*I_HEN_*_,2016_)	983.93
Total investment cost (*I_t_*_,2018_)	9036.27
Annualised capital cost	1470.61

**Table 5:** Operating cost estimates for the biorefinery process (Equations (20)–(26)).

Cost Component	Estimated Value ($kUS)
Chemical cost	109.68
Labour cost	718.08
Overhead cost	93.08
Depreciation	903.6
Total utility cost ^a^	239.94
Repair and maintenance cost	542.20
Total operating cost	2606.58

^a^ Includes the cost of cooling, heating and electricity.

**Table 6:** TMPS feed rate for optimal NER and unit production cost for case A and case B.

Cases	TMPS, *m*, (tonnes/h)	NER	*C_m_* ($US/tonne)
Case A: electricity generated from fossil sources	144.6	1.013	56.2
Case B: electricity generated from renewable sources	140.8	1.063	57.0

**Table 7:** Mass flow rate of useful products from the proposed biorefinery system.

Biorefinery System	Production Cost (US$/year)	Mass Flow Rate of Valuable Products (tonnes/h)			
		Biogas	Biocrude	Biodiesel	Biochar
Case A	5291962.85	2.698	1.241	0.734	8.405
Case B	5226156.72	2.627	1.208	0.715	8.184

**Table 8:** Mass flow rate of useful products from the proposed biorefinery system.

Biorefinery System	Unit Production Cost	Biogas	Biocrude	Biodiesel	Biochar
Case A	US$/kg	0.2724	0.5923	1.0009	0.0874
	US$/L	0.00031	0.5610	0.870	0.0446
Case B	US$/kg	0.2763	0.6008	1.0152	0.0887
	US$/L	0.00032	0.5690	0.8824	0.0452

## Supplementary Materials

The following are available online at https://www.mdpi.com/1996-1944/12/7/1030/s1, Table S1: The major costing components calculated for different feedstock mass feed rate scenarios, Table S2: Energy input, energy potential and mass flow rate of useful products generated for different feedstock mass feed rate scenarios, Table S3: The major costing components calculated for different TMPS mass feed rates, Table S4: Energy input, energy potential and mass flow rate of useful products generated for different TMPS mass feed rates for case A and Case B.
