# Supplementary material for: Techno-Economic Assessment of a Scaled-Up Meat Waste Biorefinery System: A Simulation Study
Source: Materials (Basel). 2019 Mar 28;12(7):1030. doi: 10.3390/ma12071030 (PMC6479899; doi:10.3390/ma12071030)
Supplement: Supplementary file 1 [file materials-12-01030-s001.pdf]

materials

MDPI

Supplementary Materials

The major costing components and energy flows for different feedstock mass feed rate scenarios.

Table S1. The major costing components calculated for different feedstock mass feed rate scenarios.

Mass Feed Rate (tonnes/h)			Capital Cost ($kUS/year)		Operating Cost ($kUS/year)						Unit Production Cost ($US/tonne)
DAF Sludge Waste	SY Waste	TMPS	Investment	Annualised	R&M [a]	Chemicals	Overhead	Depreciation	Utility [b]	Labour [c]	C_m
20.8	21.2	42	6684.7	1087.9	401.1	52	75.91	668.5	131.70	718.1	114.5
27.8	28.4	56.2	7403.2	1204.8	444.2	71.1	81.35	740.3	168.66	718.1	93.7
34.8	35.5	70.3	8209.4	1336.0	492.6	90.4	87.20	820.9	204.96	718.1	81.9
41.7	42.5	84.2	9036.3	1470.6	542.2	109.7	93.08	903.6	239.94	718.1	74.4
48.8	49.8	98.6	9635.0	1568.0	578.1	129.4	97.90	963.50	276.25	718.1	67.5
55.8	56.9	112.7	10491.4	1707.4	629.5	149.1	103.93	1049.1	311.42	718.1	63.6
62.5	63.7	126.2	10876.6	1770.1	652.6	169.2	107.52	1087.7	344.69	718.1	59.0

[a]: Repair and maintenance, [b]: cooling, electricity and heating cost, [c]: number of workers assumed to remain unchanged, TMPS denotes the total meat processing waste stream.

Table S2. Energy input, energy potential and mass flow rate of useful products generated for different feedstock mass feed rate scenarios.

Mass Feed Rate (tonnes/h)			Mass Flow Rate of the Useful Products (tonnes/h)				Energy Flows (GJ/h)			NER	
DAF Sludge Waste	SY Waste	TMPS	Biodiesel	Biogas	Biocrude	Biochar	Heat Demand	Electrical Demand	Chemical Energy Potential	Case A	Case B
20.8	21.2	42	0.21	0.78	0.36	2.45	32.3	0.97	38.04	1.002	1.059
27.8	28.4	56.2	0.28	1.04	0.48	3.27	43.11	1.24	50.84	1.006	1.061
34.8	35.5	70.3	0.36	1.31	0.60	4.09	53.95	1.50	63.65	1.008	1.062
41.7	42.5	84.2	0.43	1.56	0.72	4.89	64.48	1.76	76.15	1.010	1.063
48.8	49.8	98.6	0.5	1.83	0.85	5.74	75.56	2.03	89.26	1.011	1.063
55.8	56.9	112.7	0.57	2.1	0.97	6.56	86.36	2.28	102.06	1.013	1.064
62.5	63.7	126.2	0.64	2.35	1.08	7.34	96.64	2.53	114.28	1.014	1.064

Table S3. The major costing components calculated for different TMPS mass feed rates.

Designation	Mass Feed Rate (tonnes/h)	Capital Cost ($kUS/year)		Operating Cost ($kUS/year)						Unit Production Cost ($US/tonne)
	TMPS	Investment	Annualised	R&M [a]	Chemicals	Overhead	Depreciation	Utility [b]	Labour [c]	C_m
A	161.5	12763.02	2077.12	765.78	227.23	121.26	1276.3	430.77	718.1	53.39
B	242.3	16315.54	2655.28	978.93	303.24	148.75	1631.55	625.29	718.1	44.76
C	282.7	18061.72	2939.46	1083.70	389.95	162.29	1806.17	721.51	718.1	42.48

[a]: Repair and maintenance, [b]: cooling, electricity and heating cost [c]: number of workers assumed to remain unchanged, TMPS denotes the total meat processing waste stream.

Table S4. Energy input, energy potential and mass flow rate of useful products generated for different TMPS mass feed rates for case A and Case B.

Designation	Mass Feed Rate (tonnes/h)	Mass of Useful Products (tonnes/h)				Energy Flows (GJ/h)			NER	
	TMPS	Biodiesel	Biogas	Biocrude	Biochar	Heat Demand	Electrical Demand	Chemical Energy Potential	Case A	Case B
A	161.5	0.82	3	1.39	9.4	124.37	0.88	131.75	1.010	1.059
B	242.3	1.23	4.51	2.08	14.1	191.04	1.27	197.43	0.988	1.033
C	282.7	1.44	5.26	2.43	16.45	223.89	1.47	230.67	0.986	1.030
